# Molecular insights of Gas6/TAM in cancer development and therapy

**DOI:** 10.1038/cddis.2017.113

**Published:** 2017-03-23

**Authors:** Guiling Wu, Zhiqiang Ma, Wei Hu, Dongjin Wang, Bing Gong, Chongxi Fan, Shuai Jiang, Tian Li, Jianyuan Gao, Yang Yang

**Affiliations:** 1 Department of Thoracic and Cardiovascular Surgery, Affiliated Drum Tower Hospital of Nanjing University Medical School, 321 Zhongshan Road, Nanjing, Jiangsu 210008, China; 2 Department of Geriatrics, Xijing Hospital, The Fourth Military Medical University, 127 Changle West Road, Xi'an 710032, China; 3 Department of Biomedical Engineering, The Fourth Military Medical University, 169 Changle West Road, Xi'an 710032, China; 4 Department of Thoracic Surgery, Tangdu Hospital, The Fourth Military Medical University, 1 Xinsi Road, Xi'an 710038, China; 5 Department of Aerospace Medicine, The Fourth Military Medical University, 169 Changle West Road, Xi'an 710032, China

## Abstract

Since growth arrest-specific gene 6 (Gas6) was discovered in 1988, numerous studies have highlighted the role of the Gas6 protein and its receptors Tyro3, Axl and Mer (collectively referred to as TAM), in proliferation, apoptosis, efferocytosis, leukocyte migration, sequestration and platelet aggregation. Gas6 has a critical role in the development of multiple types of cancers, including pancreatic, prostate, oral, ovarian and renal cancers. Acute myelocytic leukaemia (AML) is a Gas6-dependent cancer, and Gas6 expression predicts poor prognosis in AML. Interestingly, Gas6 also has a role in establishing tumour dormancy in the bone marrow microenvironment and in suppressing intestinal tumorigenesis. Numerous studies regarding cancer therapy have targeted Gas6 and TAM receptors with good results. However, some findings have suggested that Gas6 is associated with the development of resistance to cancer therapies. Concerning these significant effects of Gas6 in numerous cancers, we discuss the roles of Gas6 in cancer development in this review. First, we introduce basic knowledge on Gas6 and TAM receptors. Next, we describe and discuss the involvement of Gas6 and TAM receptors in cancers from different organ systems. Finally, we highlight the progress in therapies targeting Gas6 and TAM receptors. This review presents the significant roles of Gas6 in cancers from different systems and may contribute to the continued promotion of Gas6 as a therapeutic target.

## Facts

- Gas6/TAM has important roles in the development of multiple types of cancer, including AML, ALL, schwannoma, glioma, thyroid carcinoma, ovarian carcinoma, lung cancer, gastric cancer, prostate cancer, renal cell carcinoma, breast cancer and melanoma.
- In general, Gas6/TAM promotes cancer advancement, and the expression of Gas6 and TAM consistently predicts poor prognosis.
- Methods targeting Gas6 and TAM receptors have been reported to suppress tumour progression, which may be a focus in clinical research.

## Open questions

- Gas6/TAM axis is not only involved in the process of tumour development but also has inhibitory roles in tumour metastasis. Based on these complex mechanisms, how do scientists design drugs that target Gas6/TAM to treat various tumours with the fewest side effects?
- Is it suitable to combine current cancer therapies and Gas6/TAM inhibitors to increase the effectiveness of these therapies?
- What are the underlying mechanisms of inhibitory role of Gas6/TAM in intestinal tumours?
- What are the clinical settings in which therapeutically targeting Gas6 may be successfully applied?

Cancer remains a deadly disease and the second leading cause of mortality worldwide.^1^ The inheritance of mutated genes and somatic mutations are major causes for the development of cancer. Numerous studies have focused on critical roles of Gas6 in cancer, and Gas6 overexpression has recently been reported in several types of human cancers, including colon, thyroid, breast, lung carcinomas, ovarian cancer and others.^2, 3, 4, 5, 6^ AML is a Gas6-dependent cancer, and Gas6 expression predicts poor prognosis in AML.^7^ Intriguingly, Gas6 inhibits intestinal cancer development.^8^ These studies indicate the complicated implication of Gas6 in cancer.

Gas6 was initially discovered in 1988 through a screen for genes that arrested the growth of embryonic mouse fibroblasts in which the expression of Gas6 was upregulated.^9^ In 1993, Manfioletti *et al.*^10^ sequenced Gas6 and reported that it is a 75 kDa secreted protein with a Gla domain at the N terminus that, based on its structure, belongs to the vitamin K-dependent (VKD) protein family. Gas6 binds receptor tyrosine kinases (RTKs) of the TAM family, which comprises Tyro3, Axl and Mer, and then activates downstream signalling through which Gas6 exerts biological effects, including cell survival, migration and growth.^11^ Moreover, Gas6 has a critical role in the development of multiple types of cancers, including pancreatic, prostate, oral, ovarian and renal cancers.^12, 13, 14, 15, 16^ Clinically, the expression of Gas6 and TAM receptors always predicts poor prognosis. In addition, Gas6 is clearly involved in cancer invasion. Targeting Gas6 and TAM can be an effective treatment strategy, as Gas6- and TAM-targeted treatments have reduced tumour progression.^17, 18, 19, 20^ Altogether, these findings provide a rationale for the continued development of Gas6- and TAM-targeted therapies. In this review, we will introduce the basic knowledge on Gas6 and the critical effects of Gas6 on cancer, and we will provide future directions and unresolved questions regarding Gas6.

## Gas6 and TAM Receptors

### Background knowledge of Gas6

**Figure 1:**
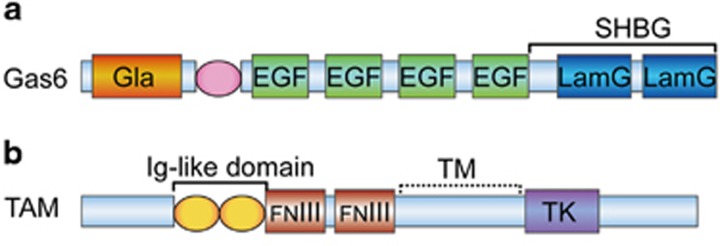
The structures of Gas6 and TAM. (**a**) The Gla domain is at the N terminus and is followed by a loop that is maintained by a disulphide bridge. Next to the loop are four EGF-like domains. At the C terminus, Gas6 contains an SHBG-like domain comprising two subdomains with similar structures to the globular motifs of LamG. (**b**) The TAM receptors, Axl, Mer and Tyro3, are RTKs that have an intracellular tyrosine kinase domain. The extracellular domains contain a combination of two N-terminal immunoglobulin (Ig)-like domains and two fibronectin type-III (FNIII) repeats. EGF, epidermal growth factor; FNIII, fibronectin type-III; Ig, immunoglobulin; LamG, globular modules of laminin G; SHBG, sex hormone-binding globulin

Gas6 is a 75 kDa secreted protein with a Gla domain at the N terminus that, based on its structure, belongs to the VKD protein family.^10^ The Gla domain is generated by VKD *γ*-carboxylation of a cluster of Glu residues and is believed to be involved in intramolecular and intermolecular protein–protein or protein–membrane interactions through Ca^2+^ binding.^21^ Moreover, the Gla domain targets Gas6 to apoptotic or activated cells that occur in a wide range of pathologies.^22^ The Gla domain of Gas6 is followed by a loop maintained by a disulphide bridge. The structure next to the loop contains four epidermal growth factor (EGF)-like domains.^10^ Each of the four EGF-like domains in Gas6 contains a consensus sequence for the *β*-hydroxylation of Asp and Asn residues,^23^ whose presence is correlated with a high-affinity Ca^2+^ binding site and seems to be involved in protein–protein interactions.^24^ Rees *et al.*^24^ showed that the EGF-like domains of Gas6 may have a role in stabilizing an active conformation of Gas6 or modulating its activity. At the C terminus, Gas6 contains a sex hormone-binding globulin (SHBG)-like domain comprising two subdomains with similar structures to the globular modules of laminin A (LamG).^10^ This type of globular structure is usually found in proteins that interact with heparin sulphates, steroids or integrins.^25^ LamG is a type of matrix protein that can interact with cell surface receptors and cause cell spreading (see Figure 1a).^26^ Gas6 binds RTKs of the TAM family and then activates downstream signalling, through which Gas6 exerts its biological effects.^11^ It is critical to determine which domain of Gas6 is essential for its receptor-binding and biological activities. Intriguingly, Mark *et al.*^27^ found that Gas6 binds receptors through an SHBG-like domain. Tanabe *et al.*^28^ reported that a Gas6 mutant composed only of an SHBG-like domain retained both its receptor-binding and biological activities.

### Background knowledge on TAM receptors

Gas6 binds to TAM receptors and then exerts pleiotropic effects in a variety of cells.^29^ The TAM receptors, Axl, Mer and Tyro3, are RTKs with an intracellular tyrosine kinase domain. The extracellular domains contain a combination of two N-terminal immunoglobulin (Ig)-like domains and two fibronectin type-III (FNIII) repeats (see Figure 1b). Gas6 stimulates TAM receptors and subsequently affects cell biological activity through downstream signalling pathways. However, Axl, Mer and Tyro3 have different affinities for Gas6, various expression patterns, different downstream signalling and diverse functions (see Table 1).

#### Axl receptor

**Figure 2:**
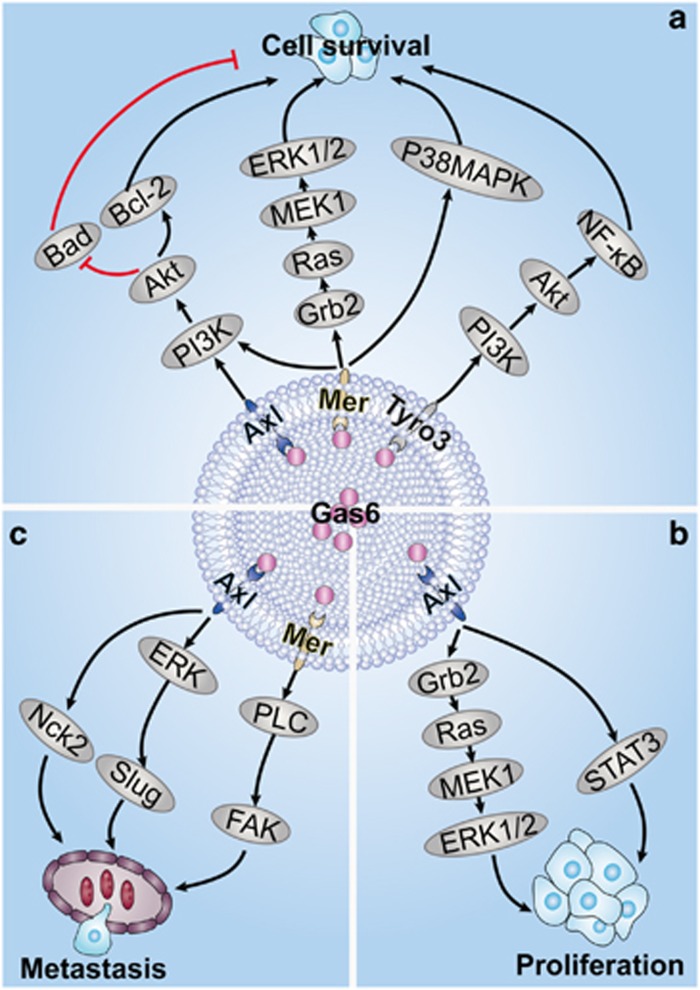
Gas6 and TAM receptor signalling. Gas6 binds to TAM and thereby exerts its biological effects, including the promotion of survival, proliferation and migration in several cancers. (**a**) Gas6/Axl interaction activates the PI3K/Akt pathway and promotes the survival of cancer cells. Activation of Akt leads to inactivation of Bad and an increase in the antiapoptotic protein Bcl-2 via an NF-*κ*B-dependent mechanism. Gas6/Mer interacts with Grb2 and promotes survival through Ras, MEK1 and upregulation of ERK1/2. Gas6/Mer also activates P38 MAPK to promote survival, and Gas6 binding at the cell surface induces dimerization and autophosphorylation of Tyro3 at its intracellular domain, which provides docking sites for downstream signalling molecules. Then, the Akt survival pathway is activated, resulting in the nuclear translocation of NF-*κ*B and the upregulation of NF-*κ*B target genes, which have a role in survival. (**b**) Gas6/Axl promote proliferation through interacting with Grb2, STAT3 and MAPK/ERK. The Gas6/Grb2 interaction can induce cell proliferation by activating Ras/ERK signalling. (**c**) Gas6/Axl interacts with Nck2, and Nck2 connects Axl to a ternary complex consisting of the PINCH protein, ILK and parvin, which is associated with migration. Gas6/Axl also induces migration through upregulation of Slug by ERK, and Gas6/Mer activation induces upregulation of FAK through PLC, which promote cell migration. Akt, protein kinase B; Bcl-2, B-cell lymphoma 2; ERK, extracellular signal-regulated kinase; FAK, focal adhesion kinase; Gas6, growth arrest-specific gene 6; Grb2, growth factor receptor-bound protein 2; ILK, integrin-linked kinase; MEK1, mitogen-activated protein kinase kinase; MAPK, mitogen-activated protein kinase; Nck2, non-catalytic region of tyrosine kinase adaptor protein 2; NF-*κ*B, nuclear factor kappa-light-chain-enhancer of activated B cells; PI3K, phosphatidylinositol 3-kinase; PINCH, particularly interesting new cysteine-histidine-rich protein; PLC, Phospholipase C; STAT, signal transducer and activator of transcription

Axl was first identified in 1991 as a product of a transforming gene in a T-cell leukaemia cell line.^37^ Axl is a 140 kDa protein ubiquitously expressed in cell lines of epithelial, mesenchymal and haematopoietic origins, as well as in non-transformed cells.^38^ Gas6 can activate a series of different signalling pathways after binding to Axl and can regulate multiple cellular functions, especially cell survival, proliferation and migration (see Figure 2).^39, 40, 41, 42^ The phosphatidylinositol 3-kinase (PI3K)-protein kinase B (Akt) pathway is a critical target of Gas6 and Axl interactions in cellular survival.^43, 44^ Activation of Akt leads to the inactivation of Bad, a proapoptotic mediator, and the increase of the antiapoptotic protein B-cell lymphoma 2 (Bcl-2) by a nuclear factor kappa-light-chain-enhancer of activated B cells (NF-*κ*B)-dependent mechanism.^45^ The interaction between Gas6 and Axl also induces cell mitogenesis through signal transducer and activator of transcription 3 (STAT3) signalling and extracellular signal-regulated kinase (ERK) signalling. The interaction of Axl–Nck2 may connect Axl to a ternary complex consisting of the particularly interesting new cysteine-histidine-rich protein (PINCH) protein, integrin-linked kinase (ILK) and parvin, which is a signalling platform at focal adhesions that regulates cytoskeletal dynamics.^46^ Based on a study by Wickström *et al.*,^47^ the hypothesis that ILK is a kinase has remained controversial. These authors showed that the proposed kinase activity of ILK does not exist and that ILK functions as a mediator of the integrin–actin linkage in cells.^47^ PINCH is a widely expressed and evolutionarily conserved protein that primarily consists of five LIM domains. LIM domain-containing proteins are composed of two contiguous zinc finger domains separated by a two-amino-acid residue hydrophobic linker, and these proteins have roles in protein–protein interactions and in the regulation of cytoskeletal organization, organ development and oncogenesis. Interestingly, Prager-Khoutorsky *et al.*^48^ proposed that the different stages of cell polarization are regulated by multiple tyrosine kinase-dependent molecular checkpoints, including Axl, that jointly control cell contractility and focal adhesion-mediated mechanosensing. In summary, Gas6 binds to Axl and induces cell survival, proliferation and migration (see Figure 2).^30, 43, 44, 45, 49^

#### Mer receptor

Mer was identified in 1994 as the chicken proto-oncogene c-eyk, which was derived from the avian retrovirus ribosomal protein L30 (RPL30).^50^ At 18–20 kDa, the Mer protein was first identified in monocytes, epithelial tissue and reproductive tissue, and thus, it received the name Mer.^51^ As an RTK, Mer is ubiquitously expressed in multiple tissues and cell types. Among peripheral blood cells, Mer is detected in monocytes and platelets but not in normal lymphocytes and neutrophils.^51^ Mer is involved in many cellular functions, including the phagocytosis of apoptotic cells and the production of cytokines.^52^ Most importantly, Mer promotes cancer progression.^53^ The upregulation of Mer in AML indicates its significance in the progression of AML.^7^ Phospholipase c-*γ* (PLC-*γ*), PI3K, Grb2, Raf-1 and ERK are phosphorylated downstream of Mer (see Figure 2).^54, 55^ Todt *et al.*^52^ have shown that PLC-*γ* is associated with Mer and at least partly responsible for the phagocytosis of apoptotic thymocytes by Mer-expressing macrophages. Furthermore, Mer can also activate the STAT transcription factor pathway. For example, stimulation of Gas6/Mer results in activation of the Janus tyrosine kinase (JAK)/STAT signalling pathway in melanoma.^56^ A microarray analysis demonstrated that Mer is a strong inducer of chemokines such as interleukin (IL)-8 in human prostate cancer cells.^57^ Wu *et al.*^58^ found that Mer activation, either with or without Gas6, induces Src-mediated tyrosine phosphorylation of focal adhesion kinase (FAK) and its recruitment to the integrin *αβ*5. FAK is a focal adhesion-associated protein kinase involved in cellular adhesion and spreading processes. When FAK is blocked, breast cancer cells become less metastatic due to decreased mobility. Integrin *αβ*5 also has a role in cell migration. To summarize, Mer is involved in cancer cell survival and migration.

#### Tyro3 receptor

Tyro3 was first identified in 1993 and was primarily found in the central nervous system (CNS).^59^ Although information on the status of Tyro3 expression in human cancer is scant, Tyro3 has been detected in several human leukaemia cell lines and blasts of acute myeloid leukaemia patients^60^ and is overexpressed in myeloma cells compared with its expression in autologous B-lymphoblastoid cell lines.^61^ Tyro3, a receptor tyrosine kinase, transduces signals from the extracellular matrix into the cytoplasm by binding to Gas6. Ligand binding at the cell surface induces dimerization and autophosphorylation of Tyro3 at its intracellular domain, which provides docking sites for downstream signalling molecules (see Figure 2).^62, 63^ Subsequently, the Akt survival pathway is activated, the nuclear translocation of NF-*κ*B is induced, and the target genes of NF-*κ*B are upregulated. Through Akt/NF-*κ*B signalling, Tyro3 exerts prosurvival effects and promotes cancer cell survival (see Figure 2). Thus, the Tyro3 receptor regulates cell survival and is involved in the progression of several cancers.^60^ Several studies have shown that Tyro3 is significantly upregulated in thyroid cancer cells and melanoma cells,^64, 65^ but few studies have examined the importance of Tyro3 overexpression. Further studies are needed in the future.

## Role of Gas6/TAM in Different Organ System

### Gas6 in cancers of the locomotor system

In the locomotor system, Gas6/Axl has been shown involved in osteosarcoma progress.^66^ Specifically, osteosarcoma, the most common histological form of primary bone cancer,^67^ is a type of aggressive malignant neoplasm that arises from primitive transformed cells of mesenchymal origin (and thus a sarcoma), exhibits osteoblastic differentiation and produces malignant osteoid.^68^ Expression of Axl has been detected in osteosarcoma tissues with a high reactivity rate compared with adjacent non-cancerous tissues,^69^ and Axl expression has been shown to be significantly correlated with recurrence and lung metastasis in osteosarcoma patients.^66^ High expression of activated Axl is an independent predictor of a worse prognosis in osteosarcoma.^66^ In the osteosarcoma cell lines MG63 and U2OS, recombinant human Gas6 (rhGas6) can cause a remarkable increase in phosphorylated-Axl (p-Axl) expression. In both cell lines, Axl activation by rhGas6 can protect tumour cells from apoptosis caused by serum starvation and promote tumour cell migration and invasion *in vitro*. Knockdown of Axl inhibits the proliferation and induces apoptosis of osteosarcoma cells, possibly through the downregulation of the Akt pathway. In addition, a strong positive correlation between p-Axl and matrix metalloproteinase (MMP)-9 expression was confirmed in these osteosarcoma patients,^66^ and proteins of the MMP family are involved in the breakdown of extracellular matrix associated with metastasis.^70^ All these results implicate Gas6/Axl in osteosarcoma, and further basic and clinical studies are needed to investigate whether the inhibition of Gas6/Axl could lead to positive results.

### Gas6 in cancers of haematological systems

In the haematological system, Gas6/TAM receptors have been shown to be involved in the development of acute leukaemia.^31, 71, 72, 73, 74^ Gas6/TAM expression is mostly observed in AML and ALL.^31, 71, 74^ More specifically, overexpression of Axl mRNA and protein and Mer protein is observed in AML patients and AML cell lines.^71, 75^ The Mer protein was found to be abnormally expressed in approximately half of paediatric T-cell leukaemia patient samples and T-ALL cell lines.^76^ However, few studies have examined the expression of Tyro3 in leukaemia. Unlike the expression of Axl and Mer, Gas6 expression is low in AML cells, similar to the situation in healthy haematopoietic cells.^75^ In contrast, Gas6 is abundant in AML bone marrow (BM) stromal cells with a fibroblastic/mesenchymal morphology (referred to as BMDSCs), whereas its expression is lower in control BMDSCs.^75^ Therefore, the mechanism of Gas6/TAM overexpression in acute leukaemia remains unclear. Interestingly, in an *in vitro* study, Ben-Batalla *et al.*^71^ found that M-CSF and IL-10, mediated by AML, instruct BMDSCs to secret Gas6.^75^ Notably, overexpression of Gas6/TAM is associated with an adverse prognosis in AML.^71, 74^ It has been shown that patients expressing Gas6 (Gas6+), especially those with ages ⩾60 years, more often fail to achieve complete remission (CR) compared with those who do not express Gas6. In all patients, Gas6+ patients exhibit shorter disease-free (DFS) and overall survival (OS) than those patients without Gas6 expression (Gas6−).^74^ In addition, patients expressing Axl at levels above the median value show significantly shorter OS than patients expressing Axl levels below the median.^71^ To investigate the function of Gas6/TAM expression, researchers have inhibited Gas6/TAM signalling using a short-hairpin RNA (shRNA) or a specific inhibitor as well as in animal experiments. For example, the clinically applicable small-molecule Axl kinase inhibitor BGB324 (formerly named R428) inhibits the phosphorylation of Axl in AML cells. Application of BGB324 monotherapy inhibits the proliferation of FLT3-mutated MV4-11 cells.^71^ When two independent shRNA constructs were used to decrease Mer expression in the AML cell lines Nomo-1 and Kasumi-1,^7^ the reduction of Mer protein levels significantly increased the rate of myeloblast apoptosis by two- to three-fold in response to serum starvation. NOD-SCID-gamma mice transplanted with Nomo-1 myeloblasts with reduced levels of Mer exhibited significant prolongation of survival compared with mice transplanted with the parental or control cell lines.^7^ These results indicate that Axl and the Mer kinase receptor promote the survival or proliferation of AML cells. Significantly, a paracrine effect of Gas6 might be required for Axl activation in AML cell lines, as Gas6 is abundant in AML bone marrow (BM) stromal cells. AML cells instruct BMDSCs to upregulate Gas6, which fosters their growth and chemoresistance.^71^ The functional significance of Gas6/TAM was demonstrated through the pharmacologic inhibition of the Gas6/TAM signalling pathway. However, genetic approaches may be required to render these findings more definitive. There are few studies on the genetic upstream signalling and genetic multiplication of Gas6/TAM. However, several studies have indicated downstream signalling involved in the biological effects of Gas6/TAM.^7, 31, 74, 76^ Whitman *et al.*^74^ derived a Gas6-associated gene-expression signature in GAS6+ patients who included overexpression of BAALC and MN1 (known to confer an adverse prognosis in AML) as well as CXCL12 (encoding a stromal cell-derived factor) and its receptor genes, chemokine (C-X-C motif) receptor 4 (CXCR4) and CXCR7. Another study showed that following the stimulation of an AML cell line with Gas6, prosurvival and proliferative signalling pathways were activated, including the phosphorylation of ERK1/2, p38, mitogen- and stress-active protein kinase 1 (MSK1), cAMP-response element binding protein (CREB), ATF-1, Akt and STAT6.^76^ In conclusion, all these results indicate that the expression of Gas6/TAM promotes the development of acute leukaemia.

### Gas6 in cancers of the nervous system

In the nervous system, the functions of Gas6/TAM in schwannomas and gliomas have been extensively researched.^32, 59, 77, 78, 79^ Schwannomas are homogeneous tumours consisting only of Schwann cells.^77^ Gliomas arise from glial cells and originate in the brain or spine. Glioblastoma multiforme (GBM) is a malignant astrocytoma constituting one type of glioma.^80^ Several studies have indicated that Gas6/TAM is overexpressed in schwannomas and gliomas.^32, 59, 77, 78, 79^ Specifically, Axl, Tyro3, Mer and their ligand Gas6 are expressed at higher levels in human primary schwannoma compared with those in normal Schwann cells.^77^ In addition, high expression levels of Axl mRNA have been found in the majority of the tested glioma cell lines, and a Northern blot analysis demonstrated coordinated expression of Axl and Gas6 mRNA in the majority of glioma cell lines.^32^ Immunohistochemical staining with an anti-Axl antibody revealed abundant Axl protein expression in glioma cells. Significantly, Axl and Gas6 are frequently overexpressed in both glioma and vascular cells and predict a poor prognosis in GBM patients.^32^ The genetic mechanisms of Gas6/TAM overexpression are not clear. However, the biological functions of Gas6/TAM in schwannoma and glioma have been demonstrated through the inhibition of Gas6/TAM signalling. For example, Vajkoczy *et al.*^79^ revealed that Axl promotes glioma growth and invasion by introducing a truncated form of human Axl lacking the intracellular RTK-bearing domain into SF126 cells (Axl-DN). To determine whether Axl signalling is relevant to tumour growth, these authors performed subcutaneous xenografts in nude mice. Compared with control cells and Axl-WT cells, the tumorigenicity of Axl-DN cells was found to be dramatically impaired. Vajkoczy *et al.*^79^ obtained similar results *in vitro* studies, showing that the overexpression of Axl-DN conferred a 50% or 30% growth disadvantage relative to mock or Axl-WT cells, respectively. Another study revealed that the downstream signalling pathway of Gas6/Axl includes Src, FAK and NF-*κ*B. NF-*κ*B regulates the Gas6/Axl-mediated overexpression of survivin, cyclin D1 and FAK, leading to the proliferation of schwannoma cells, cell-matrix adhesion and enhanced survival.^77^

### Gas6 in cancers of the endocrine system

Within the endocrine system, involvement of Gas6/TAM in thyroid cancer has been demonstrated.^3, 33, 64^ Thyroid cancer originates from follicular or parafollicular thyroid cells. These cells give rise to both well-differentiated cancers and anaplastic thyroid cancer. Expression of Gas6, Axl and Tyro3 is observed in thyroid cancer according to relevant studies.^3, 33, 64^ Avilla *et al.*^64^ found that Tyro3 and Axl are significantly upregulated and activated in thyroid cancer cells. Tyro3 and Axl protein levels are undetectable in normal thyroid cells, whereas they show significant expression in cancer cell lines,^64^ which was also confirmed in RT-polymerase chain reaction (RT-PCR) experiments. Many thyroid cancer cell lines (850-5C, NIM and CAL62) have been demonstrated to express the Axl ligand Gas6.^64^ In addition, this study showed that human thyroid cancer specimens express Axl and Gas6.^64^ Specifically, Axl positivity was observed primarily in tumoral cells, while tumoural stroma and nontumoral adjacent tissues were negative. Most of the analysed specimens were scored as positive for Gas6. Although Gas6 staining was cytosolic and primarily found in carcinoma cells, some samples also displayed stromal positivity, suggesting that the ligand can also be provided by the tumour microenvironment. Preliminary data failed to show gene mutations or amplifications in thyroid cancer cell lines, and thus, other possible mechanisms of Axl expression in thyroid cancer must be investigated.^64^ Inhibition of Tyro3, Axl or Gas6 reduced cell proliferation and increased the rate of apoptosis. Axl silencing in 850-5C ATC cells dramatically reduced the invasive ability of thyroid cancer cells.^64^ *In vivo*, Avilla *et al.*^64^ selected 850-5C cells based on their ability to induce tumour formation in immunodeficient mice. They stably transfected these cells with a pool of vectors expressing five different shRNAs directed against Axl or vectors expressing control nontargeting shRNAs and found that Axl silencing inhibited experimental tumour growth. These results suggested that Gas6/Axl and Tyro3 promote the development of thyroid cancer and that targeting Gas6/Axl and Tyro3 may offer a novel therapeutic approach for this cancer.^64^

### Gas6 in cancers of the respiratory system

Within the respiratory system, the functions of Gas6/TAM in lung cancer have been extensively investigated.^6, 34, 36, 81, 82^ The two main types are SCLC and NSCLC.^83^ Wimmel *et al.*^6^ have shown that Axl is expressed in approximately 60% of NSCLC cell lines and in normal bronchial epithelial cells (NHBE) but not in SCLC cell lines. Expression of the Axl ligand, Gas6, was detected in approximately 80% of the investigated cell lines. As NHBEs also expresses Axl, these authors suggested that the observed absence of Axl expression in SCLC tumour cells is an aberrant feature. Linger *et al.*^36^ evaluated 88 human NSCLC tumours that were of diverse histology and identified Mer and Axl overexpression in 69 and 93% of the tumours, respectively, relative to that in the surrounding normal lung tissue. Mer and Axl were also found to be frequently overexpressed and activated in NSCLC cell lines.^36^ Interestingly, combining protein expression analysis of CD68 and Gas6 with tumour (T), lymph node (N) and metastasis (M), using Cox regression or ISIR, improves the prediction of NSCLC.^34^ In addition, increased expression of Axl, as well as its ligand Gas6 in some cases, was found in EGFR-mutant lung cancers obtained from patients with acquired EGFR tyrosine kinase inhibitor (TKI) resistance.^82^ Wimmel *et al.*^6^ found that shRNA knockdown of Mer or Axl significantly reduced NSCLC colony formation and the growth of subcutaneous xenografts in nude mice. Mer or Axl knockdown also improved *in vitro* NSCLC sensitivity to chemotherapeutic agents by promoting apoptosis. Significantly, when the effects of Mer and Axl knockdown were compared, Mer inhibition was found to achieve a more complete blockade of tumour growth, while Axl knockdown improved chemosensitivity more robustly.^6^ These results indicate that Axl and Mer can promote the survival of NSCLC and enhance chemoresistance. These authors also showed that ligand-dependent Mer or Axl activation stimulated the mitogen-activated protein kinase (MAPK), Akt and FAK signalling pathways, indicating roles for Mer and Axl in multiple oncogenic processes.^6^ Presently, there has been little research on the roles of Tyro3 in lung cancer.

### Gas6 in cancers of the digestive system

In the digestive system, Gas6 is involved in OSCC, gastric cancer, PDA, intestinal tumours and hepatocellular carcinoma.^8, 13, 15, 84, 85, 86^ Gas6/Axl is detected in OSCC, and Axl can be a prognostic marker for OSCC.^13^ Jiang *et al.*^87^ explored the level and clinical significance of serum Gas6 in patients with OSCC and found that Gas6 increases the metastatic capacity of OSCC cells and that serum Gas6 could be a biomarker for diagnostic and prognostic use in OSCC patients. Gas6 expression is significantly associated with lymph node metastasis in gastric cancer tissues and cell lines.^86^ The combination of Mer expression with Axl expression inversely correlates with patient prognosis.^88^ In a study analysing the expression of Gas6 in PDA, Gas6 and Axl were frequently overexpressed in PDA cells and associated with poor prognosis in patients with stage II PDA.^15^ Gas6 and Axl promote the progression of hepatocellular carcinoma by enhancing the expression of the EMT-inducing transcription factor Slug, which is essential for the invasion-promoting activity of Axl.^85^ Interestingly, a study revealed an inhibitory role of Gas6 *in vivo* during the progression of intestinal tumours that was associated with the suppression of stromal immune reactions.^8^ In that study, compared with Gas6^+/+^ mice, Gas6^−/−^ mice exhibited enhanced azoxymethane/dextran sulphate sodium (DSS)-induced tumorigenesis and had shorter survival. Gas6^−/−^ mice also exhibited more severe DSS-induced colitis.

### Gas6 in cancers of the urinary system

Accumulating studies have indicated that Gas6 is critical for the progression of prostate cancer and renal cell carcinoma in the urinary system.^12, 14, 16, 89, 90, 91, 92, 93^ The Gas6–Axl interaction promotes mitogenic activity in undifferentiated metastatic human prostatic cancer cell lines by inducing the phosphorylation of Akt and MAPK.^14^ Bone is the preferred metastatic site of advanced prostate cancer. A study investigated the molecular basis of dormancy in the bone marrow microenvironment and found that in an osseous environment, a human prostate cancer cell line grew significantly better in vertebral body transplants derived from the Gas6^−/−^ animals than in those derived from the Gas6^+/+^ animals. These results indicate that Gas6 may be the molecular basis of bone marrow dormancy.^94^ In addition, the binding of prostate cancer to annexin II induces the expression of the Gas6 receptors Axl and Mer, which induce dormancy in the haematopoietic system.^90^ How disseminated tumour cells (DTCs) become dormant in the marrow and how dormant DTCs escape dormancy remain unclear. Interestingly, Taichman *et al.*^42^ found that when Tyro3 expression exceeds Axl expression, the prostate cancer cells exhibit rapid growth. However, when the expression of Axl predominates, prostate cells remain largely quiescent.^42^ These findings suggest that a balance between the expression of Axl and Tyro3 is associated with a molecular switch between a dormant and a proliferative phenotype in prostate cancer metastases.^42^ Similarly, the differential expression of Axl and Gas6 in renal cell carcinoma reflects tumour advancement and survival.^12^ Serum levels of soluble Axl and Gas6 protein and Gas6 mRNA level correlate with survival, metastasis and disease severity. However, no correlation between Axl protein expression in the tumour tissue and survival has yet been found.

### Gas6 in cancers of the reproductive system

In the reproductive system, Gas6, Axl and Tyro3 mRNA levels are significantly higher in uterine leiomyoma than in normal uterine myometrium.^95, 96^ Gas6 expression is evaluated in ovarian tumour tissues and is higher in tumours from patients with residual disease compared with those without residual disease. Therefore, Gas6 can be an independent potential biomarker for ovarian cancer both at the mRNA and protein levels.^97^ It has been suggested that Gas6, Axl and Tyro3 signal transduction is aberrantly stimulated in uterine leiomyoma and could possibly be related to tumour growth. Gas6/TAM also has a critical role in ovarian carcinoma.^97^ Therapeutically, the promotion of growth and invasion by Gas6 can be repressed by sodium butyrate in ovarian carcinoma cells.^35, 97^

### Gas6 in other cancers

Gas6/TAM have critical roles in other cancers, such as breast cancer and melanoma.^40, 56, 65, 98, 99, 100, 101^ Gas6 is overexpressed and amplified in breast cancer and can be upregulated by progesterone via progesterone receptor B (PRB).^99^ By using quantitative real-time PCR analysis of the levels of Gas6 mRNA expression in 49 primary breast carcinomas and evaluating the expression of the PRB protein immunohistochemically with a commercially available PRB antibody, one study showed a positive association between the PRB protein and Gas6 mRNA levels. Clinically, the level of Gas6 mRNA correlates positively with a number of favourable prognostic variables in breast cancer, including lymph node metastasis negativity, younger age at diagnosis, smaller size of tumours, lower Nottingham prognostic index scores and lower nuclear morphology score.^99^ Metastatic melanoma is one of the most aggressive forms of cutaneous cancers. Melanoma cell lines have relatively high expression of TAM receptors compared with normal cells, and TAM receptors have oncogenic properties in melanomas.^56, 65, 100^ However, the mechanisms underlying melanoma development induced by the different receptors vary. Gas6 induces Tyro3 phosphorylation and downstream Akt phosphorylation without apparent effects on ERK.^65^ In contrast, the stimulation of melanoma cells with Gas6 results in the activation of several downstream signalling pathways, including MAPK/ERK, PI3K/Akt and JAK/STAT.^56^

In summary, numerous studies have shown that Gas6 promote development of cancers from different systems (see Table 2), and targeting Gas6 therapy in the future can be more feasible.

## Targeting Gas6 and Receptors in Cancer Therapy

As noted above, Gas6/TAM has a significant role in the development of numerous cancer types,^6, 13, 33, 66, 72, 77^ highlighting Gas6/TAM as attractive targets for therapeutic development. Numerous researches considering shRNA knockdown of TAM receptors have been shown effective in inhibition of different kinds of tumours including breast carcinoma, melanoma, PDA, osteosarcoma, NSCLC, thyroid cancer and AML.^6, 7, 15, 56, 64, 69, 102^ In addition, selective small-molecule inhibitors of Axl and Mer have been generated. R428, a potent and selective Axl inhibitor blocks Axl-dependent events, including Akt phosphorylation, breast cancer cell invasion and proinflammatory cytokine production.^18^ R428 is now in clinical development. Several ongoing controlled trials involving R428 at various clinical centres are registered at ClinicalTrails.gov (Identifier: NCT02922777, NCT02488408, NCT02424617 and NCT02872259), which are aimed at identifying its maximum tolerated dose. These studies include trials of R428 in NSCLC, AML and metastatic melanoma. All of these trials are underway, and their results are expected (see Table 3). UNC1062, a novel Mer-selective small-molecule tyrosine kinase inhibitor, has been shown to reduce Mer-mediated downstream signalling activation, induce apoptosis in culture, reduce colony formation in soft agar and inhibit the invasion of melanoma cells.^56^ Moreover, an improved Mer-selective small-molecule tyrosine kinase inhibitor, UNC2025, has been shown to exert antitumour effects in GBM lines.^19^ Intriguingly, although there is no existing research on Gas6-specific inhibitors, a recent study has exploited a novel way to inhibit Gas6/Axl signalling, which may inspire the development of better cancer therapies.^103^ The authors engineered an Axl 'decoy receptor' that binds Gas6 with a high affinity to inhibit its function, allowing effective sequestration of Gas6 and specific abrogation of Axl signalling. Moreover, the increased Gas6-binding affinity is critical and correlates with the ability of decoy receptors to potently inhibit metastasis and disease progression *in vivo*. The results suggest a novel method for inhibiting Gas6/Axl signaling.^103^

## Conclusions

In earlier studies, Gas6/TAM was shown to promote cell survival, aggregation, migration and growth. Recently, the role of Gas6 in various cancers has become clearer. Gas6/TAM is involved in the development of many cancers, including AML, ALL, schwannoma, glioma, thyroid carcinoma, ovarian carcinoma, lung cancer, gastric cancer, prostate cancer, renal cell carcinoma, breast cancer and melanoma. Gas6 generally promotes cancer advancement. Clinically, the expression of Gas6 and TAM consistently predicts poor prognosis. Altogether, these findings provide a rationale for the continued development of Gas6-targeted therapies.

Numerous experimental studies on Gas6- and TAM-targeted treatments have shown reduced tumour progression, almost all of which have been conducted *in vitro*. In the future, more studies should focus on *in vivo* and clinical research. The methods targeting Gas6 and TAM receptors will become more diversified and will involve shRNA or siRNA knockdown, the use of monoclonal antibodies and classic drugs found to target Gas6.

## Figures and Tables

**Table 1:** TAM receptors

**Receptor**	**Affinity with Gas6**	**Expression patterns**	**Downstream signaling**	**Functions**	**Reference no.**
Axl	Highest affinity with Gas6	Axl is ubiquitously expressed including cell lines of epithelial, mesenchymal and haematopoietic origins, as well as non-transformed cells and also overexpressed in a wide variety of human cancers including colon, lung carcinomas, oesophageal, thyroid, breast cancers.	Gas6/Axl/PI3K/Akt pathway Gas6/Axl/MAPK/ERK pathway Gas6/Axl/STAT3 pathway Gas6/Axl also interacts with Grb2, Nck2, RanBPM, C1-TEN, SOCS1	Cell survival, proliferation, migration and mitogenesis	^38, 39, 40, 41, 42, 43, 44, 45, 46, 49^
Mer	Less affinity than Axl with Gas6	Mer was expressed in monocytes and epithelial and reproductive tissues, and also expressed in AML.	PLC-*γ*, PI3K, Grb2, Raf-1 and ERK were phosphorylated downstream of Mer. Mer also activates STAT1.	Cell migration	^51, 54, 55, 56, 57, 58^
Tyro3	Less affinity than Axl with Gas6	Tyro3 is expressed in several human leukaemic cell lines and is also overexpressed in myeloma cells.	Gas6/Tyro3/Akt/NF-*κ*B pathway Gas6/Tyro3/STAT1 pathway	Cell survival, migration and differentiation	^59, 60, 61, 62, 63, 64, 65^

Abbreviations: Akt, protein kinase B; AML, acute myeloid leukaemia; C1-TEN, C1 domain-containing phosphatase and tensin homologue; ERK, extracellular signal-regulated kinase; Gas6, growth arrest-specific gene 6; Grb2, growth factor receptor-bound protein 2; MAPK, mitogen-activated protein kinase; Nck2, non-catalytic region of tyrosine kinase adaptor protein 2; NF-*κ*B, nuclear factor kappa-light-chain-enhancer of activated B cells; PI3K, phosphatidylinositol 3-kinase; PLC-*γ*, phospholipase C *γ*; RanBPM, Ran binding protein in microtubule organizing centre; SOCS1, suppressor of cytokine signalling 1; STAT1, signal transducer and activator of transcription 1; STAT3, signal transducer and activator of transcription 3

**Table 2:** Effects of Gas6 in various cancers

**System**	**Tumour type**	**Effect of Gas6 on tumour development**	**Underlying mechanism**	**Reference no.**
Locomotor system	Osteosarcoma	Promotion	Akt activation	^66^
Circulatory system	Acute myeloid leukaemia	Promotion	ERK1/2, P38, MSK1 and CREB phosphorylation ATF-1, Akt and STAT6 activation	^75^
	Acute lymphoblastic leukaemia	Promotion	Binding of Gas6 to Mer	^76^
Nervous system	Schwannoma	Promotion	Src, FAK and NF-*κ*B recruitment	^77^
	Glioma	Promotion	Binding of Gas6 to Axl	^32^
Endocrine system	Thyroid carcinoma	Promotion	Gas6-Tyro3/Axl autocrine circuit	^64^
Respiratory system	Lung cancer	Promotion	Binding of Gas6 to Axl	^6^
Digestive system	Oral squamous cell carcinoma	Promotion	Binding of Gas6 to Axl	^13^
	Gastric cancer	Promotion	Binding of Gas6 to Axl	^86^
	Pancreatic ductal adenocarcinoma	Promotion	Akt activation	^15^
	Intestinal tumour	Inhibition		^8^
	Hepatocellular carcinoma	Promotion	Slug expression	^85^
Urinary system	Prostate cancer	Promotion	Akt and MAPK phosphorylation	^14^
	Renal cell carcinoma	Promotion	Binding of Gas6 to Axl	^12^
Reproductive system	Uterine leiomyoma	Promotion	Binding of Gas6 to Axl	^96^
	Ovarian carcinoma	Promotion	Binding of Gas6 to TAM	^97^
Other cancers	Breast cancer	Promotion	Expression of PRB and Gas6	^99^
	Melanoma	Promotion	MAPK/ERK, PI3K/Akt, JAK/STAT activation	^56^

Abbreviations: Akt, protein kinase B; ATF-1, activating transcription factor-1; CREB, cAMP-response element binding protein; ERK, extracellular signal-regulated kinase; FAK, focal adhesion kinase; Gas6, growth arrest-specific gene 6; JAK, Janus tyrosine kinase; MAPK, mitogen-activated protein kinase; MSK1, mitogen-and stress-active protein kinase 1; NF-*κ*B, nuclear factor kappa-light-chain-enhancer of activated B cells; PI3K, phosphatidylinositol 3-kinase; PRB, progesterone receptor B; STAT, signal transducer and activator of transcription; STAT1, signal transducer and activator of transcription 1

Gas6/TAM has a critical role in oncogenesis in many tissues, and most studies indicate that Gas6 promotes cancer. Interestingly, one study revealed a unique *in vivo* inhibitory role of Gas6 during the progression of intestinal tumours. The underlying mechanisms are mostly clear

**Table 3:** The ongoing clinical trials on R428 in cancers

**Identifiers**	**Start date**	**Cancers**	**Time frame**	**Phase**	**Status**
NCT02922777	November 2016	Non-small cell lung carcinoma	42 days	Phase 1	Recruiting
NCT02488408	September 2014	Acute myeloid leukaemia	15 months	Phase 1	Recruiting
NCT02424617	March 2015	Non-small cell lung cancer	3 months	Phase 1/2	Recruiting
NCT02872259	September 2016	Melanoma	An average of 1 year	Phase 1/2	Not yet recruiting

Ongoing clinical trials of R428 in various cancer types

## References

[bib1] Siveen KS, Sikka S, Surana R, Dai X, Zhang J, Kumar AP et al. Targeting the STAT3 signaling pathway in cancer: role of synthetic and natural inhibitors. Biochim Biophys Acta 2014; 1845: 136–154. 24388873 10.1016/j.bbcan.2013.12.005

[bib2] Craven RJ, Xu LH, Weiner TM, Fridell YW, Dent GA, Srivastava S et al. Receptor tyrosine kinases expressed in metastatic colon cancer. Int J Cancer 1995; 60: 791–797. 7896447 10.1002/ijc.2910600611

[bib3] Ito T, Ito M, Naito S, Ohtsuru A, Nagayama Y, Kanematsu T et al. Expression of the Axl receptor tyrosine kinase in human thyroid carcinoma. Thyroid 1999; 9: 563–567. 10411118 10.1089/thy.1999.9.563

[bib4] Meric F, Lee WP, Sahin A, Zhang H, Kung HJ, Hung MC. Expression profile of tyrosine kinases in breast cancer. Clin Cancer Res 2002; 8: 361–367. 11839650

[bib5] Sun W, Fujimoto J, Tamaya T. Coexpression of Gas6/Axl in human ovarian cancers. Oncology 2004; 66: 450–457. 15452374 10.1159/000079499

[bib6] Wimmel A, Glitz D, Kraus A, Roeder J, Schuermann M. Axl receptor tyrosine kinase expression in human lung cancer cell lines correlates with cellular adhesion. Eur J Cancer 2001: 2264–2274. 11677117 10.1016/s0959-8049(01)00271-4

[bib7] Lee-Sherick AB, Eisenman KM, Sather S, McGranahan A, Armistead PM, McGary CS et al. Aberrant Mer receptor tyrosine kinase expression contributes to leukemogenesis in acute myeloid leukemia. Oncogene 2013; 32: 5359–5368. 23474756 10.1038/onc.2013.40 PMC3898106

[bib8] Akitake-Kawano R, Seno H, Nakatsuji M, Kimura Y, Nakanishi Y, Yoshioka T et al. Inhibitory role of Gas6 in intestinal tumorigenesis. Carcinogenesis 2013; 34: 1567–1574. 23430954 10.1093/carcin/bgt069

[bib9] Schneider C, King RM, Philipson L. Genes specifically expressed at growth arrest of mammalian cells. Cell 1988; 54: 787–793. 3409319 10.1016/s0092-8674(88)91065-3

[bib10] Manfioletti G, Brancolini C, Avanzi G, Schneider C. The protein encoded by a growth arrest-specific gene (gas6) is a new member of the vitamin K-dependent proteins related to protein S, a negative coregulator in the blood coagulation cascade. Mol Cell Biol 1993; 13: 4976–4985. 8336730 10.1128/mcb.13.8.4976-4985.1993 PMC360142

[bib11] Lemke G. Biology of the TAM receptors. Cold Spring Harb Perspect Biol 2013; 5: a009076. 24186067 10.1101/cshperspect.a009076 PMC3809585

[bib12] Gustafsson A, Bostrom AK, Ljungberg B, Axelson H, Dahlback B. Gas6 and the receptor tyrosine kinase Axl in clear cell renal cell carcinoma. PLoS ONE 2009; 4: e7575. 19888345 10.1371/journal.pone.0007575 PMC2766033

[bib13] Lee CH, Yen CY, Liu SY, Chen CK, Chiang CF, Shiah SG et al. Axl is a prognostic marker in oral squamous cell carcinoma. Ann Surg Oncol 2012; 19((Suppl 3)): S500–S508. 21842265 10.1245/s10434-011-1985-8

[bib14] Sainaghi PP, Castello L, Bergamasco L, Galletti M, Bellosta P, Avanzi GC. Gas6 induces proliferation in prostate carcinoma cell lines expressing the Axl receptor. J Cell Physiol 2005; 204: 36–44. 15605394 10.1002/jcp.20265

[bib15] Song X, Wang H, Logsdon CD, Rashid A, Fleming JB, Abbruzzese JL et al. Overexpression of receptor tyrosine kinase Axl promotes tumor cell invasion and survival in pancreatic ductal adenocarcinoma. Cancer 2011; 117: 734–743. 20922806 10.1002/cncr.25483 PMC4403266

[bib16] Yamashita S, Takahashi S, McDonell N, Watanabe N, Niwa T, Hosoya K et al. Methylation silencing of transforming growth factor-beta receptor type II in rat prostate cancers. Cancer Res 2008; 68: 2112–2121. 18381416 10.1158/0008-5472.CAN-07-5282

[bib17] Ekyalongo RC, Mukohara T, Funakoshi Y, Tomioka H, Kataoka Y, Shimono Y et al. TYRO3 as a potential therapeutic target in breast cancer. Anticancer Res 2014; 34: 3337–3345. 24982338

[bib18] Holland SJ, Pan A, Franci C, Hu Y, Chang B, Li W et al. R428, a selective small molecule inhibitor of Axl kinase, blocks tumor spread and prolongs survival in models of metastatic breast cancer. Cancer Res 2010; 70: 1544–1554. 20145120 10.1158/0008-5472.CAN-09-2997

[bib19] Sufit A, Lee-Sherick AB, DeRyckere D, Rupji M, Dwivedi B, Varella-Garcia M et al. MERTK Inhibition Induces Polyploidy and Promotes Cell Death and Cellular Senescence in Glioblastoma Multiforme. PLoS ONE 2016; 11: e0165107. 27783662 10.1371/journal.pone.0165107 PMC5081168

[bib20] Tsai WB, Long Y, Kuo MT. Gas6/Axl in arginine-starvation therapy. Oncoscience 2015; 2: 659–660. 10.18632/oncoscience.218 PMC4580048 26425646

[bib21] Nakano T, Higashino K, Kikuchi N, Kishino J, Nomura K, Fujita H et al. Vascular smooth muscle cell-derived, Gla-containing growth-potentiating factor for Ca(2+)-mobilizing growth factors. J Biol Chem 1995; 270: 5702–5705. 7890695 10.1074/jbc.270.11.5702

[bib22] Huang M, Rigby AC, Morelli X, Grant MA, Huang G, Furie B et al. Structural basis of membrane binding by Gla domains of vitamin K-dependent proteins. Nat Struct Biol 2003; 10: 751–756. 12923575 10.1038/nsb971

[bib23] Stenflo J, Lundwall A, Dahlback B. beta-Hydroxyasparagine in domains homologous to the epidermal growth factor precursor in vitamin K-dependent protein S. Proc Natl Acad Sci USA 1987; 84: 368–372. 2948188 10.1073/pnas.84.2.368 PMC304208

[bib24] Rees DJ, Jones IM, Handford PA, Walter SJ, Esnouf MP, Smith KJ et al. The role of beta-hydroxyaspartate and adjacent carboxylate residues in the first EGF domain of human factor IX. EMBO J 1988; 7: 2053–2061. 3262057 10.1002/j.1460-2075.1988.tb03045.x PMC454485

[bib25] Kleinman HK, Weeks BS, Schnaper HW, Kibbey MC, Yamamura K, Grant DS. The laminins: a family of basement membrane glycoproteins important in cell differentiation and tumor metastases. Vitam Horm 1993; 47: 161–186. 8447113 10.1016/s0083-6729(08)60446-x

[bib26] Nathan C, Sanchez E. Tumor necrosis factor and CD11/CD18 (beta 2) integrins act synergistically to lower cAMP in human neutrophils. J Cell Biol 1990; 111: 2171–2181. 1699953 10.1083/jcb.111.5.2171 PMC2116341

[bib27] Mark MR, Chen J, Hammonds RG, Sadick M, Godowsk PJ. Characterization of Gas6, a member of the superfamily of G domain-containing proteins, as a ligand for Rse and Axl. J Biol Chem 1996; 271: 9785–9789. 8621659 10.1074/jbc.271.16.9785

[bib28] Tanabe K, Nagata K, Ohashi K, Nakano T, Arita H, Mizuno K. Roles of gamma-carboxylation and a sex hormone-binding globulin-like domain in receptor-binding and in biological activities of Gas6. FEBS Lett 1997; 408: 306–310. 9188782 10.1016/s0014-5793(97)00448-1

[bib29] van der Meer JH, van der Poll T, van 't Veer C. TAM receptors, Gas6, and protein S: roles in inflammation and hemostasis. Blood 2014; 123: 2460–2469. 24596417 10.1182/blood-2013-09-528752

[bib30] Hafizi S, Alindri F, Karlsson R, Dahlback B. Interaction of Axl receptor tyrosine kinase with C1-TEN, a novel C1 domain-containing protein with homology to tensin. Biochem Biophys Res Commun 2002; 299: 793–800. 12470648 10.1016/s0006-291x(02)02718-3

[bib31] Hong CC, Lay JD, Huang JS, Cheng AL, Tang JL, Lin MT et al. Receptor tyrosine kinase AXL is induced by chemotherapy drugs and overexpression of AXL confers drug resistance in acute myeloid leukemia. Cancer Lett 2008; 268: 314–324. 18502572 10.1016/j.canlet.2008.04.017

[bib32] Hutterer M, Knyazev P, Abate A, Reschke M, Maier H, Stefanova N et al. Axl and growth arrest-specific gene 6 are frequently overexpressed in human gliomas and predict poor prognosis in patients with glioblastoma multiforme. Clin Cancer Res 2008; 14: 130–138. 18172262 10.1158/1078-0432.CCR-07-0862

[bib33] Ito M, Nakashima M, Nakayama T, Ohtsuru A, Nagayama Y, Takamura N et al. Expression of receptor-type tyrosine kinase, Axl, and its ligand, Gas6, in pediatric thyroid carcinomas around chernobyl. Thyroid 2002; 12: 971–975. 12490074 10.1089/105072502320908303

[bib34] Kossler W, Fiebeler A, Willms A, ElAidi T, Klosterhalfen B, Klinge U. Formation of translational risk score based on correlation coefficients as an alternative to Cox regression models for predicting outcome in patients with NSCLC. Theor Biol Med Model 2011; 8: 28. 21794149 10.1186/1742-4682-8-28 PMC3156745

[bib35] Krupitza G, Grill S, Harant H, Hulla W, Szekeres T, Huber H et al. Genes related to growth and invasiveness are repressed by sodium butyrate in ovarian carcinoma cells. Br J Cancer 1996; 73: 433–438. 8595156 10.1038/bjc.1996.78 PMC2074449

[bib36] Linger RM, Cohen RA, Cummings CT, Sather S, Migdall-Wilson J, Middleton DH et al. Mer or Axl receptor tyrosine kinase inhibition promotes apoptosis, blocks growth and enhances chemosensitivity of human non-small cell lung cancer. Oncogene 2013; 32: 3420–3431. 22890323 10.1038/onc.2012.355 PMC3502700

[bib37] Liu E, Hjelle B, Bishop JM. Transforming genes in chronic myelogenous leukemia. Proc Natl Acad Sci USA 1988; 85: 1952–1956. 3279421 10.1073/pnas.85.6.1952 PMC279899

[bib38] O'Bryan JP, Frye RA, Cogswell PC, Neubauer A, Kitch B, Prokop C et al. axl, a transforming gene isolated from primary human myeloid leukemia cells, encodes a novel receptor tyrosine kinase. Molecular and cellular biology 1991; 11: 5016–5031. 1656220 10.1128/mcb.11.10.5016-5031.1991 PMC361494

[bib39] Dormady SP, Zhang XM, Basch RS. Hematopoietic progenitor cells grow on 3T3 fibroblast monolayers that overexpress growth arrest-specific gene-6 (GAS6). Proc Natl Acad Sci USA 2000; 97: 12260–12265. 11050245 10.1073/pnas.97.22.12260 PMC17329

[bib40] Gjerdrum C, Tiron C, Hoiby T, Stefansson I, Haugen H, Sandal T et al. Axl is an essential epithelial-to-mesenchymal transition-induced regulator of breast cancer metastasis and patient survival. Proc Natl Acad Sci USA 2010; 107: 1124–1129. 20080645 10.1073/pnas.0909333107 PMC2824310

[bib41] Holland SJ, Powell MJ, Franci C, Chan EW, Friera AM, Atchison RE et al. Multiple roles for the receptor tyrosine kinase axl in tumor formation. Cancer Res 2005; 65: 9294–9303. 16230391 10.1158/0008-5472.CAN-05-0993

[bib42] Taichman RS, Patel LR, Bedenis R, Wang J, Weidner S, Schumann T et al. GAS6 receptor status is associated with dormancy and bone metastatic tumor formation. PLoS ONE 2013; 8: e61873. 23637920 10.1371/journal.pone.0061873 PMC3634826

[bib43] Braunger J, Schleithoff L, Schulz AS, Kessler H, Lammers R, Ullrich A et al. Intracellular signaling of the Ufo/Axl receptor tyrosine kinase is mediated mainly by a multi-substrate docking-site. Oncogene 1997; 14: 2619–2631. 9178760 10.1038/sj.onc.1201123

[bib44] Shieh YS, Lai CY, Kao YR, Shiah SG, Chu YW, Lee HS et al. Expression of axl in lung adenocarcinoma and correlation with tumor progression. Neoplasia 2005; 7: 1058–1064. 16354588 10.1593/neo.05640 PMC1501169

[bib45] Goruppi S, Ruaro E, Varnum B, Schneider C. Gas6-mediated survival in NIH3T3 cells activates stress signalling cascade and is independent of Ras. Oncogene 1999; 18: 4224–4236. 10435635 10.1038/sj.onc.1202788

[bib46] Tu Y, Li F, Wu C. Nck-2, a novel Src homology2/3-containing adaptor protein that interacts with the LIM-only protein PINCH and components of growth factor receptor kinase-signaling pathways. Mol Biol Cell 1998; 9: 3367–3382. 9843575 10.1091/mbc.9.12.3367 PMC25640

[bib47] Wickstrom SA, Lange A, Montanez E, Fassler R. The ILK/PINCH/parvin complex: the kinase is dead, long live the pseudokinase!. EMBO J 2010; 29: 281–291. 20033063 10.1038/emboj.2009.376 PMC2824469

[bib48] Prager-Khoutorsky M, Lichtenstein A, Krishnan R, Rajendran K, Mayo A, Kam Z et al. Fibroblast polarization is a matrix-rigidity-dependent process controlled by focal adhesion mechanosensing. Nat Cell Biol 2011; 13: 1457–1465. 22081092 10.1038/ncb2370

[bib49] Yanagita M, Arai H, Nakano T, Ohashi K, Mizuno K, Fukatsu A et al. Gas6 induces mesangial cell proliferation via latent transcription factor STAT3. J Biol Chem 2001; 276: 42364–42369. 11546821 10.1074/jbc.M107488200

[bib50] Jia R, Hanafusa H. The proto-oncogene of v-eyk (v-ryk) is a novel receptor-type protein tyrosine kinase with extracellular Ig/GN-III domains. J Biol Chem 1994; 269: 1839–1844. 7507487

[bib51] Graham DK, Dawson TL, Mullaney DL, Snodgrass HR, Earp HS. Cloning and mRNA expression analysis of a novel human protooncogene, c-mer. Cell Growth Differ 1994; 5: 647–657. 8086340

[bib52] Todt JC, Hu B, Curtis JL. The receptor tyrosine kinase MerTK activates phospholipase C gamma2 during recognition of apoptotic thymocytes by murine macrophages. J Leukoc Biol 2004; 75: 705–713. 14704368 10.1189/jlb.0903439 PMC2441598

[bib53] Xie S, Li Y, Li X, Wang L, Yang N, Wang Y et al. Mer receptor tyrosine kinase is frequently overexpressed in human non-small cell lung cancer, confirming resistance to erlotinib. Oncotarget 2015; 6: 9206–9219. 25826078 10.18632/oncotarget.3280 PMC4496212

[bib54] Besser D, Bromberg JF, Darnell JEJr, Hanafusa H. A single amino acid substitution in the v-Eyk intracellular domain results in activation of Stat3 and enhances cellular transformation. Mol Cell Biol 1999; 19: 1401–1409. 9891073 10.1128/mcb.19.2.1401 PMC116068

[bib55] Guttridge KL, Luft JC, Dawson TL, Kozlowska E, Mahajan NP, Varnum B et al. Mer receptor tyrosine kinase signaling: prevention of apoptosis and alteration of cytoskeletal architecture without stimulation or proliferation. J Biol Chem 2002; 277: 24057–24066. 11929866 10.1074/jbc.M112086200

[bib56] Schlegel J, Sambade MJ, Sather S, Moschos SJ, Tan AC, Winges A et al. MERTK receptor tyrosine kinase is a therapeutic target in melanoma. J Clin Invest 2013; 123: 2257–2267. 23585477 10.1172/JCI67816 PMC3639697

[bib57] Wu YM, Robinson DR, Kung HJ. Signal pathways in up-regulation of chemokines by tyrosine kinase MER/NYK in prostate cancer cells. Cancer Res 2004; 64: 7311–7320. 15492251 10.1158/0008-5472.CAN-04-0972

[bib58] Wu Y, Singh S, Georgescu MM, Birge RB. A role for Mer tyrosine kinase in alphavbeta5 integrin-mediated phagocytosis of apoptotic cells. J Cell Sci 2005; 118: 539–553. 15673687 10.1242/jcs.01632

[bib59] Lai C, Lemke G. An extended family of protein-tyrosine kinase genes differentially expressed in the vertebrate nervous system. Neuron 1991; 6: 691–704. 2025425 10.1016/0896-6273(91)90167-x

[bib60] Crosier PS, Hall LR, Vitas MR, Lewis PM, Crosier KE. Identification of a novel receptor tyrosine kinase expressed in acute myeloid leukemic blasts. Leuk Lymphoma 1995; 18: 443–449. 8528051 10.3109/10428199509059643

[bib61] De Vos J, Couderc G, Tarte K, Jourdan M, Requirand G, Delteil MC et al. Identifying intercellular signaling genes expressed in malignant plasma cells by using complementary DNA arrays. Blood 2001; 98: 771–780. 11468178 10.1182/blood.v98.3.771

[bib62] Ahtiainen L, Mirantes C, Jahkola T, Escutenaire S, Diaconu I, Osterlund P et al. Defects in innate immunity render breast cancer initiating cells permissive to oncolytic adenovirus. PLoS ONE 2010; 5: e13859. 21079774 10.1371/journal.pone.0013859 PMC2974645

[bib63] Lan Z, Wu H, Li W, Wu S, Lu L, Xu M et al. Transforming activity of receptor tyrosine kinase tyro3 is mediated, at least in part, by the PI3 kinase-signaling pathway. Blood 2000; 95: 633–638. 10627473

[bib64] Avilla E, Guarino V, Visciano C, Liotti F, Svelto M, Krishnamoorthy G et al. Activation of TYRO3/AXL tyrosine kinase receptors in thyroid cancer. Cancer Res 2011; 71: 1792–1804. 21343401 10.1158/0008-5472.CAN-10-2186

[bib65] Demarest SJ, Gardner J, Vendel MC, Ailor E, Szak S, Huang F et al. Evaluation of Tyro3 expression, Gas6-mediated Akt phosphorylation, and the impact of anti-Tyro3 antibodies in melanoma cell lines. Biochemistry 2013; 52: 3102–3118. 23570341 10.1021/bi301588c

[bib66] Han J, Tian R, Yong B, Luo C, Tan P, Shen J et al. Gas6/Axl mediates tumor cell apoptosis, migration and invasion and predicts the clinical outcome of osteosarcoma patients. Biochem Biophys Res Commun 2013; 435: 493–500. 23684620 10.1016/j.bbrc.2013.05.019

[bib67] Ottaviani G, Jaffe N. The epidemiology of osteosarcoma. Cancer Treat Res 2009; 152: 3–13. 20213383 10.1007/978-1-4419-0284-9_1

[bib68] Luetke A, Meyers PA, Lewis I, Juergens H. Osteosarcoma treatment - where do we stand? A state of the art review. Cancer Treat Rev 2014; 40: 523–532. 24345772 10.1016/j.ctrv.2013.11.006

[bib69] Zhang Y, Tang YJ, Man Y, Pan F, Li ZH, Jia LS. Knockdown of AXL receptor tyrosine kinase in osteosarcoma cells leads to decreased proliferation and increased apoptosis. Int J Immunopathol Pharmacol 2013; 26: 179–188. 23527720 10.1177/039463201302600117

[bib70] Vandooren J, Van den Steen PE, Opdenakker G. Biochemistry and molecular biology of gelatinase B or matrix metalloproteinase-9 (MMP-9): the next decade. Crit Rev Biochem Mol Biol 2013; 48: 222–272. 23547785 10.3109/10409238.2013.770819

[bib71] Ben-Batalla I, Schultze A, Wroblewski M, Erdmann R, Heuser M, Waizenegger JS et al. Axl, a prognostic and therapeutic target in acute myeloid leukemia mediates paracrine crosstalk of leukemia cells with bone marrow stroma. Blood 2013; 122: 2443–2452. 23982172 10.1182/blood-2013-03-491431

[bib72] Dirks W, Rome D, Ringel F, Jager K, MacLeod RA, Drexler HG. Expression of the growth arrest-specific gene 6 (GAS6) in leukemia and lymphoma cell lines. Leuk Res 1999; 23: 643–651. 10400186 10.1016/s0145-2126(99)00075-2

[bib73] Migdall-Wilson J, Bates C, Schlegel J, Brandao L, Linger RM, DeRyckere D et al. Prolonged exposure to a Mer ligand in leukemia: Gas6 favors expression of a partial Mer glycoform and reveals a novel role for Mer in the nucleus. PLoS ONE 2012; 7: e31635. 22363695 10.1371/journal.pone.0031635 PMC3282750

[bib74] Whitman SP, Kohlschmidt J, Maharry K, Volinia S, Mrozek K, Nicolet D et al. GAS6 expression identifies high-risk adult AML patients: potential implications for therapy. Leukemia 2014; 28: 1252–1258. 24326683 10.1038/leu.2013.371 PMC4047202

[bib75] Lee-Sherick AB, Eisenman KM, Sather S, McGranahan A, Armistead PM, McGary CS et al. Aberrant Mer receptor tyrosine kinase expression contributes to leukemogenesis in acute myeloid leukemia. Oncogene 2016; 35: 6270. 27593932 10.1038/onc.2016.295 PMC5143365

[bib76] Brandao LN, Winges A, Christoph S, Sather S, Migdall-Wilson J, Schlegel J et al. Inhibition of MerTK increases chemosensitivity and decreases oncogenic potential in T-cell acute lymphoblastic leukemia. Blood Cancer J 2013; 3: e101. 23353780 10.1038/bcj.2012.46 PMC3556576

[bib77] Ammoun S, Provenzano L, Zhou L, Barczyk M, Evans K, Hilton DA et al. Axl/Gas6/NFkappaB signalling in schwannoma pathological proliferation, adhesion and survival. Oncogene 2014; 33: 336–346. 23318455 10.1038/onc.2012.587

[bib78] Staflin K, Zuchner T, Honeth G, Darabi A, Lundberg C. Identification of proteins involved in neural progenitor cell targeting of gliomas. BMC Cancer 2009; 9: 206. 19558675 10.1186/1471-2407-9-206 PMC2713262

[bib79] Vajkoczy P, Knyazev P, Kunkel A, Capelle HH, Behrndt S, von Tengg-Kobligk H et al. Dominant-negative inhibition of the Axl receptor tyrosine kinase suppresses brain tumor cell growth and invasion and prolongs survival. Proc Natl Acad Sci USA 2006; 103: 5799–5804. 16585512 10.1073/pnas.0510923103 PMC1458653

[bib80] Reuss D, von Deimling A. Hereditary tumor syndromes and gliomas. Recent Results Cancer Res 2009; 171: 83–102. 19322539 10.1007/978-3-540-31206-2_5

[bib81] Wimmel A, Rohner I, Ramaswamy A, Heidtmann HH, Seitz R, Kraus M et al. Synthesis and secretion of the anticoagulant protein S and coexpression of the Tyro3 receptor in human lung carcinoma cells. Cancer 1999; 86: 43–49. 10391562 10.1002/(sici)1097-0142(19990701)86:1<43::aid-cncr8>3.0.co;2-d

[bib82] Zhang Z, Lee JC, Lin L, Olivas V, Au V, LaFramboise T et al. Activation of the AXL kinase causes resistance to EGFR-targeted therapy in lung cancer. Nat Genet 2012; 44: 852–860. 22751098 10.1038/ng.2330 PMC3408577

[bib83] Cooper WA, Lam DC, O'Toole SA, Minna JD. Molecular biology of lung cancer. J Thorac Dis 2013; 5((Suppl 5)): S479–S490. 24163741 10.3978/j.issn.2072-1439.2013.08.03 PMC3804875

[bib84] He L, Zhang J, Jiang L, Jin C, Zhao Y, Yang G et al. Differential expression of Axl in hepatocellular carcinoma and correlation with tumor lymphatic metastasis. Mol Carcinog 2010; 49: 882–891. 20635370 10.1002/mc.20664

[bib85] Lee HJ, Jeng YM, Chen YL, Chung L, Yuan RH. Gas6/Axl pathway promotes tumor invasion through the transcriptional activation of Slug in hepatocellular carcinoma. Carcinogenesis 2014; 35: 769–775. 24233839 10.1093/carcin/bgt372

[bib86] Sawabu T, Seno H, Kawashima T, Fukuda A, Uenoyama Y, Kawada M et al. Growth arrest-specific gene 6 and Axl signaling enhances gastric cancer cell survival via Akt pathway. Mol Carcinog 2007; 46: 155–164. 17186543 10.1002/mc.20211

[bib87] Jiang T, Liu G, Wang L, Liu H. Elevated serum Gas6 is a novel prognostic biomarker in patients with oral squamous cell carcinoma. PLoS ONE 2015; 10: e0133940. 26207647 10.1371/journal.pone.0133940 PMC4514879

[bib88] Wu CW, Li AF, Chi CW, Lai CH, Huang CL, Lo SS et al. Clinical significance of AXL kinase family in gastric cancer. Anticancer Res 2002; 22: 1071–1078. 12168903

[bib89] Gustafsson A, Martuszewska D, Johansson M, Ekman C, Hafizi S, Ljungberg B et al. Differential expression of Axl and Gas6 in renal cell carcinoma reflecting tumor advancement and survival. Clin Cancer Res 2009; 15: 4742–4749. 19567592 10.1158/1078-0432.CCR-08-2514

[bib90] Haldrup C, Mundbjerg K, Vestergaard EM, Lamy P, Wild P, Schulz WA et al. DNA methylation signatures for prediction of biochemical recurrence after radical prostatectomy of clinically localized prostate cancer. J Clin Oncol 2013; 31: 3250–3258. 23918943 10.1200/JCO.2012.47.1847

[bib91] Mishra A, Wang J, Shiozawa Y, McGee S, Kim J, Jung Y et al. Hypoxia stabilizes GAS6/Axl signaling in metastatic prostate cancer. Mol Cancer Res 2012; 10: 703–712. 22516347 10.1158/1541-7786.MCR-11-0569 PMC3378814

[bib92] Nimmagadda S, Pullambhatla M, Lisok A, Hu C, Maitra A, Pomper MG. Imaging Axl expression in pancreatic and prostate cancer xenografts. Biochem Biophys Res Commun 2014; 443: 635–640. 24333418 10.1016/j.bbrc.2013.12.014 PMC3918901

[bib93] Shiozawa Y, Pedersen EA, Patel LR, Ziegler AM, Havens AM, Jung Y et al. GAS6/AXL axis regulates prostate cancer invasion, proliferation, and survival in the bone marrow niche. Neoplasia 2010; 12: 116–127. 20126470 10.1593/neo.91384 PMC2814350

[bib94] Jung Y, Shiozawa Y, Wang J, McGregor N, Dai J, Park SI et al2012 Prevalence of prostate cancer metastases after intravenous inoculation provides clues into the molecular basis of dormancy in the bone marrow microenvironment. Neoplasia 14: 429–439. 22745589 10.1596/neo.111740 PMC3384430

[bib95] Sun WS, Fujimoto J, Tamaya T. Coexpression of growth arrest-specific gene 6 and receptor tyrosine kinases Axl and Sky in human uterine endometrial cancers. Ann Oncol 2003; 14: 898–906. 12796028 10.1093/annonc/mdg257

[bib96] Sun WS, Fujimoto J, Tamaya T. Clinical implications of coexpression of growth arrest-specific gene 6 and receptor tyrosine kinases Axl and Sky in human uterine leiomyoma. Mol Hum Reprod 2003; 9: 701–707. 14561812 10.1093/molehr/gag082

[bib97] Buehler M, Tse B, Leboucq A, Jacob F, Caduff R, Fink D et al. Meta-analysis of microarray data identifies GAS6 expression as an independent predictor of poor survival in ovarian cancer. Biomed Res Int 2013; 2013: 238284. 23878800 10.1155/2013/238284 PMC3710590

[bib98] Gujral TS, Karp RL, Finski A, Chan M, Schwartz PE, MacBeath G et al. Profiling phospho-signaling networks in breast cancer using reverse-phase protein arrays. Oncogene 2013; 32: 3470–3476. 22945653 10.1038/onc.2012.378 PMC3670968

[bib99] Mc Cormack O, Chung WY, Fitzpatrick P, Cooke F, Flynn B, Harrison M et al. Growth arrest-specific gene 6 expression in human breast cancer. Br J Cancer 2008; 98: 1141–1146. 18283315 10.1038/sj.bjc.6604260 PMC2275480

[bib100] Sensi M, Catani M, Castellano G, Nicolini G, Alciato F, Tragni G et al. Human cutaneous melanomas lacking MITF and melanocyte differentiation antigens express a functional Axl receptor kinase. J Invest Dermatol 2011; 131: 2448–2457. 21796150 10.1038/jid.2011.218

[bib101] van Ginkel PR, Gee RL, Shearer RL, Subramanian L, Walker TM, Albert DM et al. Expression of the receptor tyrosine kinase Axl promotes ocular melanoma cell survival. Cancer Res 2004; 64: 128–134. 14729616 10.1158/0008-5472.can-03-0245

[bib102] Janning M, Ben-Batalla I, Loges S. Axl inhibition: a potential road to a novel acute myeloid leukemia therapy? Expert Rev Hematol 2015; 8: 135–138. 25578023 10.1586/17474086.2015.997704

[bib103] Kariolis MS, Miao YR, Jones DS2nd, Kapur S, Mathews II, Giaccia AJ et al. An engineered Axl 'decoy receptor' effectively silences the Gas6-Axl signaling axis. Nat Chem Biol 2014; 10: 977–983. 25242553 10.1038/nchembio.1636 PMC4372605

